# Sex-related differences in left atrial substrate among patients with atrial fibrillation: evidence from high-density voltage mapping

**DOI:** 10.1186/s40001-024-01952-y

**Published:** 2024-07-02

**Authors:** Wenchao Huang, Huaxin Sun, Shiqiang Xiong, Yan Luo, Yan Tang, Zhen Zhang, Hanxiong Liu

**Affiliations:** Department of Cardiology, The Third People’s Hospital of Chengdu, 82 Qinglong St, Chengdu, 610031 Sichuan China

**Keywords:** Atrial fibrillation, Sex, voltage, Ablation

## Abstract

**Background:**

There is sufficient evidence that women with atrial fibrillation (AF) have a greater symptom burden than men with AF and are more likely to experience recurrence after catheter ablation. However, the mechanisms underlying these sex differences are unclear.

**Methods:**

We prospectively enrolled 125 consecutive patients, including 40 non-AF patients and 85 AF patients, who underwent high-density voltage mapping during sinus rhythm and AF patients who underwent first ablation.

**Results:**

Overall, 37 (44%) female patients with AF and 24 (60%) female non-AF patients with a mean age of 61.7 ± 11.6 years and 53.6 ± 16.7 years, respectively, were enrolled in this study. The results showed that the atrial voltage of female AF patients was significantly lower than that of male AF patients (1.11 ± 0.58 mV vs. 1.53 ± 0.65 mV; P = 0.003), while there were no significant sex differences in non-AF patients (3.02 ± 0.86 mV vs. 3.21 ± 0.84 mV; P = 0.498). Multiple linear regression analysis revealed that female sex (− 0.29, 95% confidence interval [CI] − 0.64 to − 0.13, P = 0.004) and AF type (− 0.32, 95% CI − 0.69 to − 0.13, P = 0.004) were the only factors independently associated with voltage. Compared with men, women in the paroxysmal AF group had a 3.5-fold greater incidence of recurrence (adjusted hazard ratio 4.49; 95% CI 1.101–18.332, P = 0.036). Both globally and regionally, the results showed that sex-related differences in voltage values occurred prominently in paroxysmal AF patients but not in nonparoxysmal AF patients.

**Conclusion:**

Sex-related differences in atrial substrates and arrhythmia-free survival were found in paroxysmal AF patients, suggesting the existence of sex-related pathophysiological factors.

## Introduction

Atrial fibrillation (AF) is a common arrhythmia that can result in stroke, heart failure, dementia, and other serious complications [1]. Similar to other cardiovascular diseases, sex-based differences exist in AF patients. Men are at a greater risk of developing AF [2], and this factor can be used as an independent predictor of new-onset AF [3]. However, women with AF tend to be more symptomatic, have worse quality of life scores [4] and are more likely to experience adverse events such as stroke, heart failure, and death [5]. In addition, women have a lower rate of sinus rhythm maintenance after ablation [6, 7] and are more likely to experience postoperative adverse events [8]. Together, these studies indicate that men are more susceptible to AF, whereas women are more likely to experience adverse effects. It is essential to explore these sex-related differences to improve the diagnosis, management, and prevention of AF.

Atrial substrate remodelling is associated with the triggering and maintenance of AF [9]. Atrial fibrosis is an important manifestation of atrial substrate remodelling and can be identified and quantified using various techniques, such as cardiac magnetic resonance imaging (MRI), electroanatomical voltage mapping, and echocardiography. In addition, cardiac MRI has demonstrated a correlation between atrial fibrosis and the bipolar voltage amplitude; that is, the more severe the atrial fibrosis is, the lower the voltage [10, 11].

Although several previous studies have confirmed lower atrial voltage in women with AF [12, 13], these studies have not fully elucidated the association between biological sex and atrial voltage. Furthermore, it is unknown whether sex-related voltage differences also exist in patients without AF, implying that sex-related substrate differences are related to AF. Therefore, we performed a prespecified study to verify the association between female sex and low voltage values and the effect on long-term sinus rhythm maintenance after ablation in patients with AF.

## Methods

### Study design and population

This was a prospective single-centre observational cohort study. A total of 125 patients who underwent radiofrequency ablation at the Third People’s Hospital of Chengdu between November 2017 and December 2018 were consecutively enrolled, including 85 patients with nonvalvular AF and 40 patients with supraventricular tachycardia. The study protocol was approved by the local Ethics Committee (ChiECRCT-20170082) and was conducted in accordance with the Declaration of Helsinki and institutional guidelines. All patients signed an informed consent form for high-density voltage mapping before catheter ablation.

The inclusion criteria for the study were as follows: (1) aged ≥ 18 years; (2) diagnosed with AF or supraventricular tachycardia through 12-lead electrocardiogram or 3-lead 24-h Holter monitoring; and (3) drug-refractory patients with indications for radiofrequency ablation. The exclusion criteria were as follows: (1) history of left atrium (LA) catheter ablation or LA surgery; (2) LA thrombosis or other absolute contraindications for catheter ablation; (3) AF caused by reversible or pathological factors, including but not limited to hyperthyroidism, acute alcoholism, electrolyte disturbance, drugs, and cardiac surgery; (4) severe valvular disease; and (5) incomplete voltage data. Paroxysmal AF was defined as episodes lasting < 7 days with spontaneous termination. Nonparoxysmal AF was defined as an episode lasting ≥ 7 days or requiring drug or electroversion to terminate the AF.

### Procedural study protocol

Prior to ablation, transthoracic echocardiography was performed to determine left ventricular ejection fraction and left atrial diameter (LAD). In all patients, antiarrhythmic medications were discontinued for at least five half-lives before the procedure. LA thrombus was excluded using transoesophageal echocardiography within 48 h before the procedure. Following general anaesthesia and muscular paralysis, all catheter operations, including voltage mapping and ablation, were performed under fluoroscopic guidance and haemodynamic and electrocardiographic monitoring. The electrophysiological studies and catheter ablation were performed by two experienced operators (H.L. and Z.Z.). The activated clotting time was maintained between 300 and 350 s using intravenous unfractionated heparin.

### Electroanatomical mapping

Cardioversion was performed prior to mapping in patients who presented with AF on the day of the procedure. Patients whose sinus rhythm could not be restored were excluded from the analysis. LA geometries were created separately using a 20-polar Pentaray high-density mapping catheter (2-mm interelectrode spacing, Biosense Webster). An electrophysiological recording system (WorkMate Claris System, USA) combined with a three-dimensional electrophysiological navigation system (CARTO 3 System, Biosense Webster, USA) was used to record the intracardiac electrograms. Intracardiac electrograms were recorded using a bandpass filter set at 30–500 Hz. An internal point filter was used to ensure that the acquired voltage data were within 5 mm of the geometric surface. To minimize the mapping shift associated with respiration, a respiratory gating method was used to ensure that the anatomical points were acquired only at the end of exhalation during mechanical ventilation. The confidence module for tissue proximity indication detection was used to assess whether the tissue-catheter contact at each acquired point was sufficient for electrode-tissue contact. Considering the temporal amplitude variability of the intracardiac electrograms during acquisition, each point was selected by recording the maximal peak-to-peak bipolar voltage amplitude value within the window of interest during 10 consecutive QRS complexes (except during the QRS duration).

### Ablation strategy

Following mapping, different ablation strategies were used in patients with different AF types. In paroxysmal AF patients, the pulmonary veins were electrically isolated to achieve afferent and efferent blocking of both the pulmonary veins and the atrium. For nonparoxysmal AF patients, additional ablations were performed around the low-voltage zones, defined as an area of at least 1 cm^2^ and containing ≥ 3 neighbouring points (bipolar voltage < 0.5 mV) with ≤ 10 mm distance. To prevent stroke in AF patients, anticoagulation drugs were continued for at least 3 months. In addition, to prevent short-term AF recurrence, if there was no contraindication, oral amiodarone was administered for at least 3 months.

### Global and areal atrial voltage analysis

**Fig. 1:**
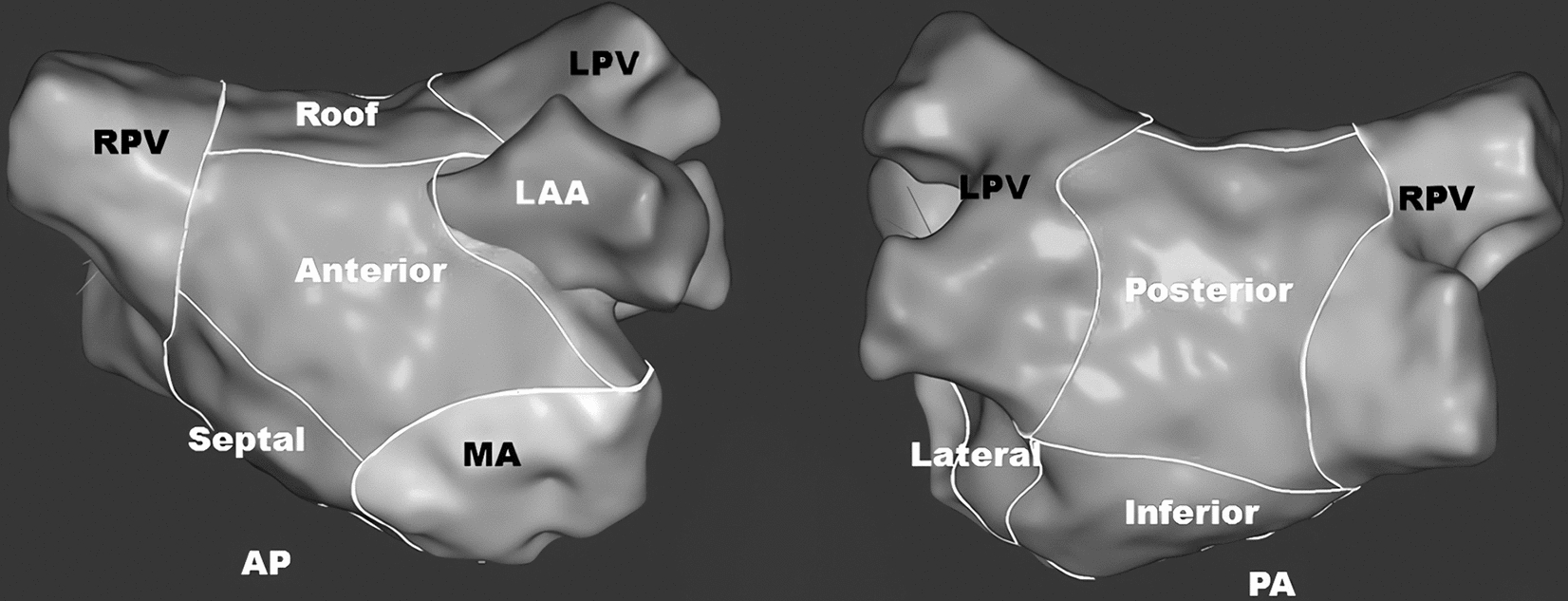
Differences in the LA geometry areas. LA geometry was divided into the following seven areas for analysis: anterior, inferior, septal, lateral, left atrial appendage, posterior and roof. *LA* left atrium

Global median left atrial voltage values were used to represent the atrial voltage value. In addition, areal voltage analysis was performed by dividing the LA geometry into seven areas: anterior, inferior, septal, lateral, left atrial appendage, posterior, and roof (Fig. 1). Points within the pulmonary veins and the mitral annulus were excluded from the analysis. The geometry was evaluated by an experienced operator (H.L.) who was blinded to the study design and carefully demarcated the different areas. A minimum of 30 voltage points were mapped and collected in each of the divided areas [14].

### Baseline and follow-up data

The baseline demographic data, clinical data, and surgical records of the patients were collected from the electronic information system of Chengdu Third People’s Hospital. All patients were followed-up regularly at 3, 6, and 12 months after ablation and annually after 1 year, with the use of 24-h Holter monitoring and questioning about any symptoms associated with arrhythmia. The patient was instructed to visit the nearest hospital for a 12-lead electrocardiogram as soon as possible if symptoms recurred after ablation. The primary follow-up endpoint was late recurrence after a single procedure, which was defined as any atrial arrhythmia (tachycardia, atrial flutter, or AF) lasting > 30 s on electrocardiography or Holter recordings that occurred > 3 months after the procedure. Follow-up results were obtained by outpatient physicians who were blinded to the study design.

### Statistical analysis

Categorical variables are presented as counts and proportions (%), and continuous variables are expressed as the mean ± standard deviation (SD) or median and interquartile range, as appropriate. Unpaired t tests, Mann–Whitney U tests, and chi-square tests (or Fisher’s exact tests), as appropriate, were used to compare differences in clinical characteristics between the sexes. Univariate linear regression analysis was used to evaluate the differential variables to determine their correlation with voltage. Variables with P < 0.1 were included in the multivariate model to determine factors independently related to voltage. The difference in voltage between the sexes was compared using an unpaired t test. Kaplan–Meier survival curves and log-rank tests were used to assess differences in the primary endpoint between men and women. Cox regression models were used to estimate the hazard ratio (HR) and associated 95% confidence interval (CI) for sex differences in AF recurrence. In addition, multiple variables were included to verify the reliability of the regression results.

All tests were two-sided, and a p value < 0.05 was considered to indicate statistical significance. Baseline, voltage and regression analyses were performed using SPSS software (version 29.0; California, USA). Kaplan–Meier survival curves and log-rank tests were performed using R software (version 4.3.1; Vienna, Austria).

## Results

### Baseline and voltage characteristics

Overall, 37 (44%) female patients with AF and 24 (60%) female non-AF patients with a mean age of 61.7 ± 11.6 years and 53.6 ± 16.7 years, respectively, were enrolled in this study. As shown in Table 1, regarding the baseline clinical characteristics of the AF patients, the female patients were older and had lower voltage values than the male patients. In addition, although the LAD was greater in men than in women, the indexed LAD was greater in women than in men, but the difference was not statistically significant. There were no significant differences in the other baseline data.

**Table 1:** Baseline characteristics of patients with AF

Parameters	Total n = 85	Females n = 37	Males n = 48	P value
Age, years	61.7 ± 11.6	65.5 ± 10.2	58.8 ± 11.8	0.007
BMI, kg/m^2^	24.9 ± 4.5	24.5 ± 5.3	25.1 ± 3.8	0.490
AF duration, months	12 (2, 48)	24 (2, 48)	12 (2, 60)	0.857
Paroxysmal AF	45 (52.9)	21 (56.8)	24 (50.0)	0.536
Previous medical history, n (%)				
Hypertension	43 (50.6)	20 (54.1)	23 (47.9)	0.575
Diabetes mellitus	13 (15.3)	7 (18.9)	6 (12.5)	0.415
CHD	15 (17.6)	5 (13.5)	10 (20.8)	0.380
CHF	14 (16.5)	7 (18.9)	7 (14.6)	0.593
Previous stroke	3 (3.5)	1 (2.7)	2 (4.2)	1.000
COPD	2 (2.4)	2 (5.4)	0 (0)	0.187
Dyslipidemia	19 (22.4)	8 (21.6)	11 (22.9)	0.887
Fasting blood glucose, mmol/L	5.34 ± 1.26	5.54 ± 1.21	5.19 ± 1.28	0.220
Triglyceride, mmol/L	1.41 ± 1.07	1.34 ± 0.84	1.46 ± 1.20	0.635
Cholesterol, mmol/L	4.09 ± 1.02	4.16 ± 1.19	4.04 ± 0.91	0.624
Albumin, g/L	39.6 ± 4.0	39.1 ± 3.8	40.1 ± 4.1	0.263
BNP, pg/mL	160 (67, 322)	201 (91, 360)	143 (49, 240)	0.104
LAD, mm	39.5 ± 6.0	38.6 ± 6.1	40.1 ± 5.9	0.249
Indexed LAD	21.6 ± 3.3	22.3 ± 3.3	21.1 ± 3.2	0.087
LVEF, %	60.1 ± 5.0	60.7 ± 4.2	59.6 ± 5.5	0.299
Atrial voltage values, mV	1.35 ± 0.65	1.11 ± 0.58	1.53 ± 0.65	0.003
Antiarrhythmic drugs, n (%)	75 (88.2)	31 (83.8)	44 (91.7)	0.320

Data presented as mean (standard deviation) for continuous variables, or n (%) for categorical variables

Indexed LAD, denotes left atrial diameter divided by body surface area

*BMI* body mass index, *CHD* coronary heart disease, *CHF* chronic heart failure, *COPD* chronic obstructive pulmonary disease, *BNP* brain natriuretic peptide, *LAD* left atrial diameter, *LVEF* left ventricular ejection fraction, *AF* atrial fibrillation

As shown in Table 2, for patients without AF, their baseline data, including atrial voltage values, were not significantly different between men and women. Similarly, men had a larger LAD than women did, but women had a larger indexed LAD than men did; however, the differences were not statistically significant. In addition, none of the patients had a history of chronic obstructive pulmonary disease or heart failure, so the data are not presented.

**Table 2:** Baseline characteristics of patients without AF

Parameters	Total n = 40	Females n = 24	Males n = 16	P value
Age, years	53.6 ± 16.7	52.6 ± 14.1	55.1 ± 20.4	0.657
BMI, kg/m^2^	23.2 ± 3.1	23.1 ± 2.6	23.2 ± 3.8	0.946
Time between symptoms and ablation, months	54 (10, 165)	48 (3, 143)	54 (15, 240)	0.234
Previous medical history, n (%)				
Hypertension	8 (20.0)	4 (16.7)	4 (25.0)	0.690
Diabetes mellitus	6 (15.0)	3 (12.5)	3 (18.8)	0.668
CHD	1 (2.5)	1 (4.2)	0 (0)	1.000
Previous stroke	1 (2.5)	0 (0)	1 (6.3)	0.400
Dyslipidemia	12 (30.0)	6 (25.0)	6 (37.5)	0.490
Fasting blood glucose, mmol/L	4.92 ± 1.01	4.88 ± 0.82	4.98 ± 1.28	0.774
Triglyceride, mmol/L	1.64 ± 0.98	1.99 ± 1.23	1.40 ± 0.70	0.119
Cholesterol, mmol/L	4.39 ± 0.93	4.45 ± 0.73	4.29 ± 1.19	0.658
Albumin, g/L	40.8 ± 4.06	40.8 ± 3.8	40.7 ± 4.6	0.958
BNP, pg/mL	97 (36, 117)	97 (43, 109)	109 (26, 138)	0.359
LAD, mm	35.1 ± 3.9	34.1 ± 3.9	36.5 ± 3.5	0.057
Indexed LAD	20.7 ± 2.5	21.0 ± 2.3	20.2 ± 2.6	0.333
LVEF, %	60.4 ± 3.4	60.4 ± 3.0	60.4 ± 4.0	0.985
Atrial voltage values, mV	3.09 ± 0.85	3.02 ± 0.86	3.21 ± 0.84	0.498
Antiarrhythmic drugs, n (%)	31 (77.5)	19 (79.2)	12 (75.0)	1.000

Data presented as mean (standard deviation) for continuous variables, or n (%) for categorical variables

Indexed LAD, denotes left atrial diameter divided by body surface area

*BMI* body mass index, *CHD* coronary heart disease, *BNP* brain natriuretic peptide, *LAD* left atrial diameter, *LVEF* left ventricular ejection fraction, *AF* atrial fibrillation

### Relationships between female sex and atrial voltage

Univariate linear regression revealed that the atrial voltage amplitude was associated with age, sex, body mass index, type of AF, diabetes mellitus, and the indexed LAD (Table 3). After adjusting for confounding factors, the results showed that female sex (standardized *β* = − 0.29, 95% CI − 0.64 to − 0.13, P = 0.004) and nonparoxysmal AF (standardized *β* = − 0.32, 95% CI − 0.69 to − 0.13, P = 0.004) were independently associated with the atrial voltage amplitude.

**Table 3:** Univariate and multivariate linear regression analysis for predicting atrial voltage values

Parameters	Univariate		Multivariate	
	*β* (95% CI)	P value	*β*^a^ (95%CI)	P value
Age	− 0.17 (− 0.03 to − 0.01)	0.004	− 0.10 (− 0.02 to 0.06)	0.338
Female sex	− 0.42 (− 0.69 to − 0.15)	0.005	− 0.29 (− 0.64 to − 0.13)	0.004
BMI	− 0.03 (− 0.61 to 0.01)	0.061	− 0.17 (− 0.05 to 0.01)	0.099
AF duration	0.00 (− 0.01 to 0.01)	0.855		
Nonparoxysmal AF	− 0.50 (− 0.76 to − 0.24)	< 0.001	− 0.32 (− 0.69 to − 0.13)	0.004
Hypertension	− 0.14 (− 0.42 to 0.14)	0.324		
Diabetes mellitus	− 0.37 (− 0.75 to 0.19)	0.062	− 0.13 (− 0.58 to 0.10)	0.165
CHD	− 0.06 (− 0.43 to 0.32)	0.766		
CHF	− 0.27 (− 0.64 to 0.11)	0.162		
Previous stroke	0.22 (− 0.54 to 0.99)	0.561		
COPD	0.01 (− 0.92 to 0.94)	0.986		
Dyslipidemia	0.09 (− 0.26 to − 0.43)	0.605		
BNP	0.00 (− 0.00 to 0.00)	0.339		
Indexed LAD	− 0.06 (− 0.11 to − 0.02)	0.003	− 0.12 (− 0.07 to 0.02)	0.317
LVEF	0.02 (− 0.01 to 00.5)	0.128		

^a^Standardized

Indexed LAD, denotes left atrial diameter divided by body surface area

*BMI* body mass index, *CHD* coronary heart disease, *CHF* chronic heart failure, *COPD* chronic obstructive pulmonary disease, *BNP* brain natriuretic peptide, *LAD* left atrial diameter, *LVEF* left ventricular ejection fraction, *AF* atrial fibrillation

### Analysis of voltage values in different groups

**Fig. 2:**
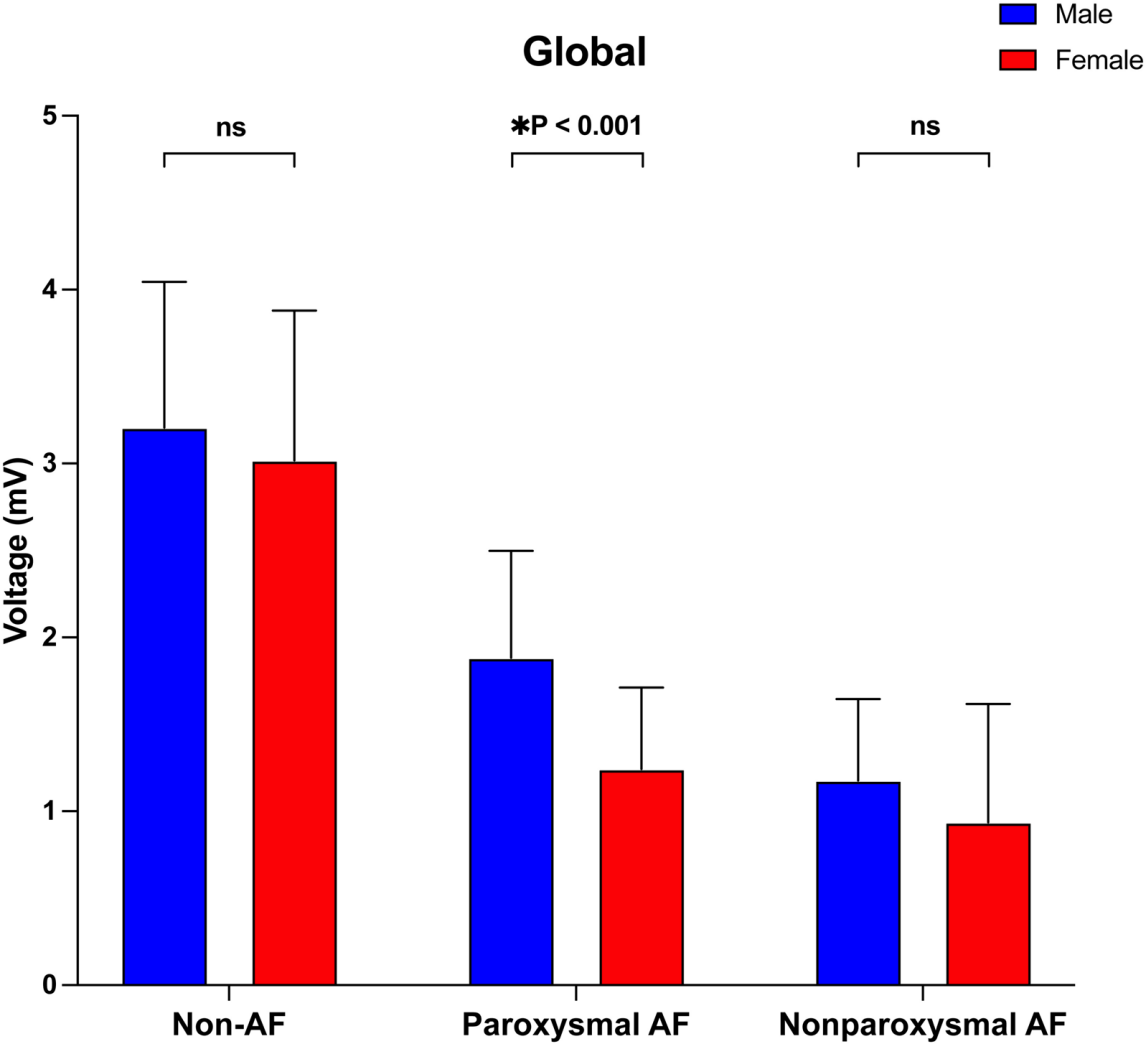
Comparison of the global voltage parameters in the different groups. *AF* atrial fibrillation

**Fig. 3:**
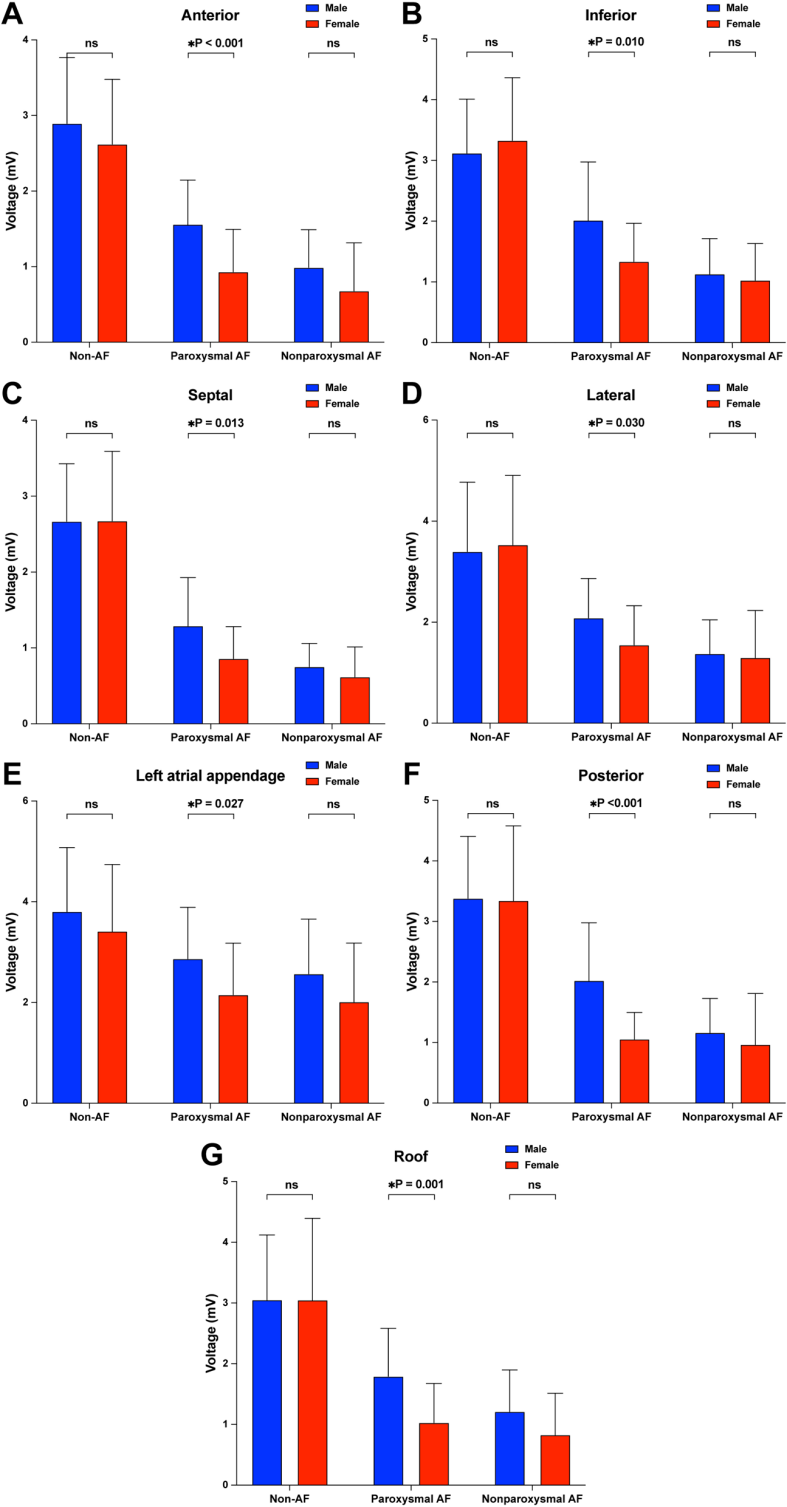
Comparison of the areal voltage parameters in the different groups. *AF* atrial fibrillation

In both men and women, the global atrial voltage decreased gradually in the non-AF cohort, the paroxysmal AF cohort and the nonparoxysmal AF cohort. There was a significant sex-related difference in the atrial voltage only in the paroxysmal AF cohort. In nonparoxysmal AF patients, although the voltage of males was still greater than that of females, there was no significant difference. Compared with non-AF females, female patients with paroxysmal AF had a significantly lower atrial voltage, which was less evident in men than in women (Fig. 2). The left atrium was divided into seven areas for analysis. Consistent with the results of the global voltage analysis, sex-related voltage differences in different atrial areas also occurred in patients with paroxysmal AF (Fig. 3). However, compared with that of the other areas, the voltage values of the left atrial appendage (Fig. 3E) decreased the most slowly across the three groups.

### Sex differences in ablation outcomes

**Fig. 4:**
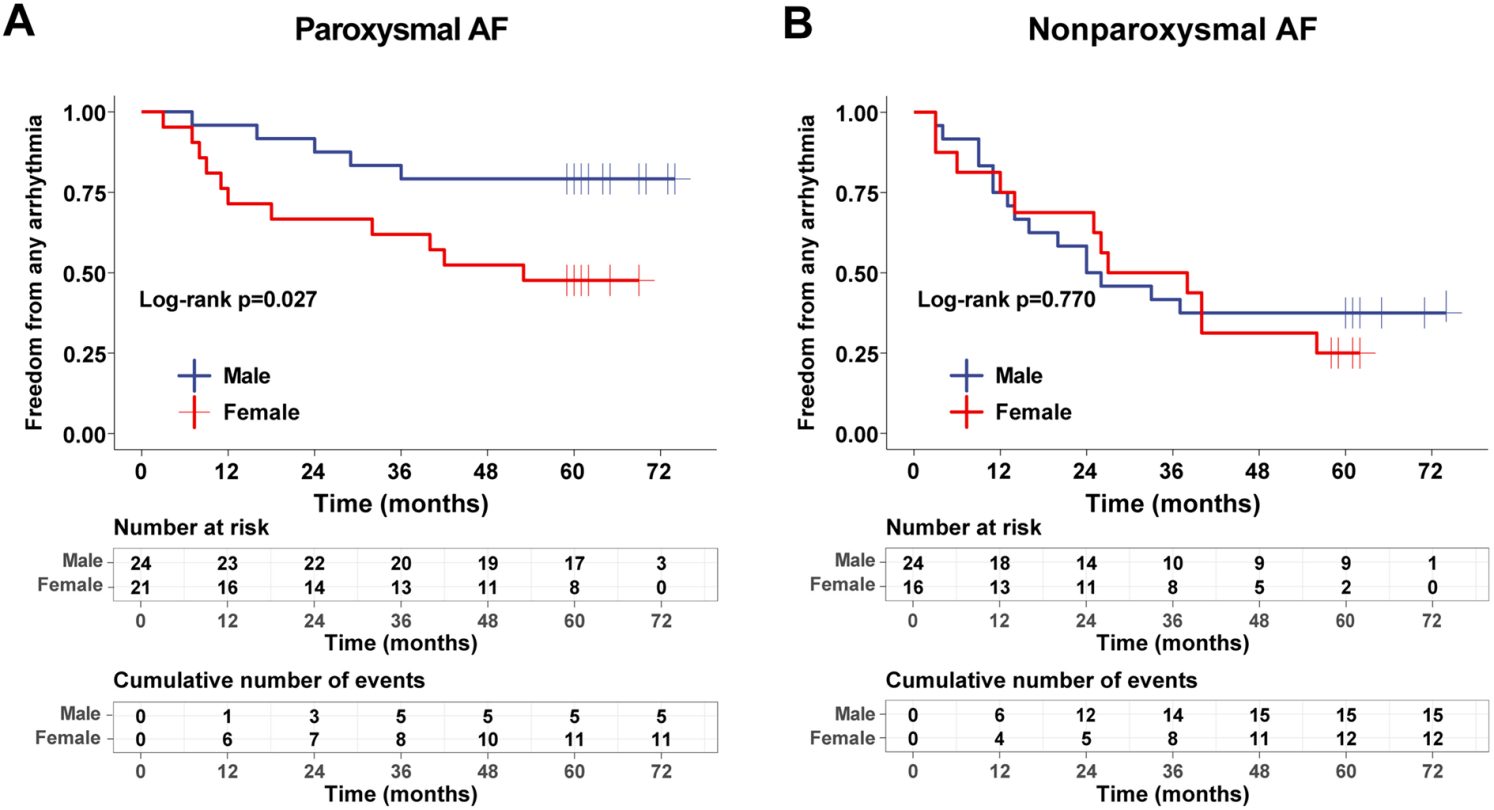
AF patients were free from any arrhythmias after a single procedure. Kaplan–Meier survival curves showing no arrhythmias after first ablation. **A** There was a statistically significant difference between men and women with paroxysmal AF. **B** There was no statistically significant difference between men and women with nonparoxysmal AF. *AF* atrial fibrillation

In the entire cohort of patients with paroxysmal AF, after a median follow-up of 62 months (interquartile range, 60 to 65 months), 29 patients (64%) were free from arrhythmias after a single procedure. Furthermore, the Kaplan–Meier survival curves showed a significant difference between men and women in terms of freedom from arrhythmia (log-rank p = 0.027) (Fig. 4A). In the entire cohort of patients with nonparoxysmal AF, after a median follow-up of 61 months (interquartile range, 60 to 65 months), 13 patients (33%) were free from arrhythmias after a single procedure. However, the Kaplan–Meier survival curves showed no significant difference in the rate of freedom from arrhythmia between men and women (log-rank p = 0.770) (Fig. 4B).

As shown in Table 4, univariate Cox regression analysis revealed that female sex was associated with a greater long-term recurrence rate in the paroxysmal AF cohort (HR = 3.099, 95% CI = 1.075–8.933; P = 0.036). Furthermore, after adjusting for relevant confounding factors, female sex was an independent predictor of long-term recurrence (HR = 4.492, 95% CI = 1.101–18.332; P = 0.036). In the nonparoxysmal AF cohort, female sex was not significantly associated with long-term recurrence (HR = 0.624, 95% CI = 0.394–4.724, P = 0.624) and was not an independent predictor of recurrence (HR = 1.167, 95% CI = 0.139–9.828, P = 0.887).

**Table 4:** Freedom from atrial tachyarrhythmia recurrence between female and male

Female	HR (95% CI)^a^	P value
Model 1		
Paroxysmal AF	3.099 (1.075–8.933)	0.036
Nonparoxysmal AF	0.624 (0.394–4.724)	0.624
Model 2		
Paroxysmal AF	4.183 (1.281–13.658)	0.018
Nonparoxysmal AF	1.913 (0.431–8.494)	0.393
Model 3		
Paroxysmal AF	4.018 (1.169–13.811)	0.027
Nonparoxysmal AF	1.540 (0.320–7.410)	0.590
Model 4		
Paroxysmal AF	4.492 (1.101–18.332)	0.036
Nonparoxysmal AF	1.167 (0.139–9.828)	0.887

^a^Results are reported as hazard ratio (95% confidence interval) for females compared to males calculated by cox regression

Model 1: unadjusted model

Model 2: adjusted for age and LAD

Model 3: adjusted for model 2 covariates plus baseline diabetes, BMI

Model 4: adjusted for model 3 covariates plus baseline hypertension, stroke, CHF, COPD and LVEF

*BMI* body mass index, *CHF* chronic heart failure, *COPD* chronic obstructive pulmonary disease, *LAD* left atrial diameter, *LVEF* left ventricular ejection fraction, *AF* atrial fibrillation

## Discussion

### Key findings

This was a prospective observational study using high-density electroanatomic mapping to investigate the presence of significant sex-related differences in atrial substrates in non-AF, paroxysmal AF, and nonparoxysmal AF patients. Our main findings were as follows: (1) there was no significant sex-related difference in atrial substrates across the non-AF patient cohort, suggesting that sex-related differences in atrial substrates are associated with AF; (2) female sex and type of AF were independent predictors of atrial voltage; (3) for paroxysmal AF, but not for nonparoxysmal AF, the atrial voltage was significantly lower in women than in men, and the incidence of no arrhythmia was significantly lower in women than in men; and (4) with the occurrence and development of AF, the voltage of each area showed a synchronous decreasing trend. This study demonstrated that the atrial voltage of female patients with AF is lower than that of male patients, which is mainly reflected in patients with paroxysmal AF, and these female patients have a high long-term recurrence rate.

### Voltage as an indicator of atrial remodelling

The progression of atrial fibrosis plays an important role in the maintenance of AF and often negatively affects the prognosis of patients receiving radiofrequency ablation [15]. In one experiment, low-amplitude abnormal bipolar electrography was shown to be associated with the ventricular scarring observed via histopathological analysis [16]. Although atrial fibrosis can be detected by noninvasive imaging methods based on delayed enhancement MRI, its application is limited in some aspects, such as being less than ideal for detecting scarring in thin-walled atria [17]. Currently, atrial bipolar voltage mapping is becoming increasingly common in substrate studies of AF. Although lower bipolar voltages help indicate atrial fibrosis, the voltage threshold for determining the fibrosis that maintains AF is not uniform [18]. The widely employed 0.5 mV voltage threshold, commonly used to identify low-voltage zones, originated from a previous study that included only 16 AF patients [19]. To avoid measurement errors, the proportion of signals was used to represent the degree of atrial remodelling instead of the size of the low-voltage zones. However, recent studies have confirmed that the atrial voltage changes over time or at different pacing cycle lengths and directions [20, 21]. Thus, it is reasonable to assume that the voltage threshold for fibrosis may vary rather than remain fixed. The atrial voltage amplitude symbolizes the general atrial activation pattern, which is the result of vector rectification and signal cancellation [22]. Therefore, the voltage was used as a substrate indicator to indirectly describe the atrium in this study rather than using ‘black or white’ values with clear boundaries. Notably, the left atrial appendage voltage decreased more slowly than that in the other areas across the three subgroups, which may be a good reference for the evaluation of lesions in other areas.

### Sex, atrial remodelling, and clinical outcomes

In an MRI study, the extent of delayed enhancement was greater in women with AF than in men with AF and was thought to represent more severe LA fibrosis [23]. Although sex differences in atrial voltage are relatively well established, these studies have not observed sex differences in AF freedom after ablation [24, 25]. In the analysis of upstream risk factors, our previous work revealed that for overall AF patients, female sex can provide additional predictive value of the APPLE score for AF recurrence [26]. This study further demonstrated a sex difference in the electroautonomic mapping data from the perspective of the AF substrates. A recent study demonstrated that older women with paroxysmal AF have more advanced atrial substrates than men, and the addition of low-voltage ablation can improve the success rate of ablation [27]. Interestingly, in this study, the use of voltage amplitudes rather than low-voltage areas also confirmed that women with paroxysmal AF had lower voltages than men did and a greater long-term recurrence rate. However, in patients with nonparoxysmal AF, the voltage was very low in both men and women, resulting in a nonsignificant difference in voltage and long-term recurrence. In a follow-up study of AF patients after cryoablation, it was also confirmed that AF freedom differences occurred in paroxysmal AF patients but not in nonparoxysmal AF patients [28]. Theoretically, electrical substrate remodelling, which is usually transient and reversible, is highly important for the maintenance of paroxysmal AF, whereas histological remodelling, which is often irreversible and progressive, is the main cause of the maintenance of paroxysmal and persistent AF [29]. It is worth noting that the duration of AF was longer in women than in men in this study, although the difference did not show statistical significance, suggesting that we should pay attention to early AF in women and that timely ablation may prevent or slow the deterioration of AF lesions. In this study, female sex was found to be an influencing factor for low atrial voltage, suggesting the potential predictive value of intraoperative voltage acquisition for the risk of postoperative recurrence of AF. These results also indirectly suggest that sex differences in the risk of recurrence may be due in part to differences in voltage values or the degree of atrial lesions.

### Mechanisms of sex-related differences in the atrium

Currently, the exact mechanism underlying sex-related differences in the impact of AF is not fully understood. A study of patients with AF who underwent multiple ablations suggested that women had a lower rate of pulmonary vein reconnection but a greater rate of long-term recurrence than men [30]. It is reasonable to infer that the lower freedom from AF in female patients after ablation is mainly related to the atrial substrate. In this study, there were no sex-related substrate differences in non-AF patients. In addition, female patients with paroxysmal AF had more significant voltage reductions than male patients, which may indicate that women have more severe early AF lesions. Compared with men, postmenopausal women with AF have more periatrial adipose tissue, which is associated with a lower atrial voltage [31]. It is worth noting that the majority of women with AF in this study were postmenopausal. Studies have shown that female hormones, including progesterone and oestrogen, affect the electrical activity of heart muscle cells [32, 33]. Another study showed that androgens significantly improve heart function in mice [34]. Taken together, these studies partially explain the advanced atrial substrates in women compared with men during early AF. However, the mechanisms underlying these differences and their clinical implications require further investigation. In addition, actively paying attention to early AF in women and timely intervention are recommended to prevent or slow the advancement of AF substrates.

### Strengths and limitations

This study revealed a potential causal relationship between low voltage and recurrence and provides new evidence for women with a high risk of recurrence. However, there are several limitations to our study. First, the sample size included in this study was relatively small. Second, regular follow-ups after ablation may have overestimated the recurrence rate, particularly in asymptomatic patients. Third, multipole catheter collection points were used; however, multipole catheters lack contact force data, potentially leading to the collection of inaccurate internal signals. Nonetheless, strict criteria were used in this study to ensure the selection of endocardial points, including manual analysis of all points and internal point filters. Fourth, right atrial mapping data were not systematically collected, and nonpulmonary venous triggers in the right atrium may play an important role in arrhythmia maintenance.

## Conclusion

In patients with paroxysmal AF, but not nonparoxysmal AF, women have more advanced atrial substrates and higher long-term recurrence rates than men. Attention should be given to early AF in women, and appropriate intervention may be an effective way to improve the treatment of AF.

## Data Availability

The original contributions presented in the study are included in the article, and further inquiries can be directed to the corresponding author/s.
